# Selections and Abstracts

**Published:** 1902-07

**Authors:** 


### SELECTIONS AND ABSTRACTS.

---

### The President's Address.*

Since the session held at St. Paul a year ago a former president of this Association, paying the last great debt to nature, has passed

> "To where beyond these voices there is peace."

Full of years and beyond the limit allotted by the Psalmist, Professor Edward Mott Moore, born in Rahway, N. J., July 15, 1814, died in Rochester, N. Y., March 3, 1902. He was one of the founders of the New York State Medical Association, a consistent and loyal friend of our national body and of an organized medical profession. Although his achievements in science were of a high order, his life was not circumscribed within the narrow limits of professional work. He was not only a skilful surgeon, bold and original, but more than this, he was a citizen of the highest type. The welfare of his neighbors, of his adopted city, State and the nation were his. May his noble example be emulated, for it is just as much our duty to be true to the obligations of citizenship as to our profession.

Before dealing with the more urgent matters of this meeting, your attention is called to the Fourteenth International Congress of Medicine, which is to be held at Madrid from April 23 to 30, 1903.

As your presiding officer I had the honor to receive an appointment from the Secretary of State, as a delegate to represent this Association at the Congress, and was requested by him to appoint five additional delegates from this body. In conforming to this request the following gentlemen have accepted commissions, and have received certificates from the State Department to the Congress: Dr. Nicholas Senn of Illinois, Dr. Maurice H. Richard-

---

* Delivered at the Fifty-third Annual Session of the American Medical Association at Sarotoga Springs, June 10–13, 1902, by John Allan Wyeth, M.D., LL.D., New York City.

son of Massachusetts, Dr. George Crile of Ohio, Dr. Richard Douglas of Tennessee, and Dr. Edward B. Dench of New York.

It should be the duty and pride of the separate State Associations to send at least one delegate to this important meeting, and in doing this to correspond with Dr. Angel Fernandez-Caro, Secretary-General, Fourteenth International Congress of Medicine, Madrid, Spain.

This session marks an era in the history of the American Medical Association, for we meet under changed conditions, and in this our trial year, while we are adjusting ourselves to the new order, we confidentially ask and expect to receive not only the consideration and forbearance, but the generous help which should be accorded to this experiment in government by which we earnestly hope to avoid the embarrassments and failures that under the old régime characterized the meetings of a body so large and unwieldy as the general session.

These changes involve not only the government of the Association proper, but also a changed relationship of the State Association to the national body, as well as the relationship of the county to the State organization. Under the old organization our business was transacted by delegates from State, district and local affiliated societies in the proportion of one delegate for each ten members, while now only affiliated State organizations have the right to send delegates, and these are only entitled to one delegate for each five hundred active members, or fraction of this number. These form the House of Delegates which is further reinforced by two members from each of the scientific sections of the Association, and one each from the Army, the Navy and the Marine Hospital Service. Under the old régime the State Association bore the same relationship to the national body as did the city, county and district organizations. Now only the State Associations are represented, and they create the legislative body of the American Medical Association. In other words, the House of Delegates is a federation of all the State Associations.

The reorganization at St. Paul, having taken away from the county organization its right to send delegates, also deprived the city, district branches and other minor associations of the same privilege, requiring membership in the county society, when such exists, as a prerequisite to membership in the State and American Medical Association. This ruling dropped, at least temporarily, from the rolls of the Association a considerable number of physicians who had long been on the roster from the State or local bodies which, by the laws then existing, were in affiliation with the National Association. While this action may have seemed unnecessary and unjust to these members (among whom are many of the most loyal and faithful supporters of the national body) for the common good, they should yield to the opinion of the majority, since calm reflection must convince any reasonable mind that one of the wisest steps the Association ever took was when it made the county medical organizations the basis of membership in the national body. There will hereafter be excluded from membership that fortunately small, but none the less existing group of unworthy members of our profession, who, on account of the clumsy rules which formerly prevailed, obtained a place on the roster of the American Medical Association.

To the date of the reorganization in June, 1901, the roster of this Association was so inaccurate and unsatisfactory that the secretary and president-elect undertook the difficult task of obtaining a correct list of members. While one might infer that each organized State and territorial association could, from its records, furnish at short notice the names of all eligible to membership in the American Medical Association, in only a very small proportion of these subordinate bodies was a reliable list available. It then became necessary to direct a circular letter, State by State, to every name on the roster then existing, asking for the necessary information. Since, for final confirmation, these names must be referred back to the subordinate organizations in which membership is claimed, it will be seen that some time must elapse before the completion of a perfectly reliable list. The lack of businesslike methods with which our profession is charged is in a manner sustained by this admission, and it emphasizes the necessity of a thorough reorganization of all the societies in affiliation with the national body, upon practically a uniform plan.

Scarcely second in importance to a uniform scheme of reorganization is that of a uniform standard of requirements for the practice of medicine in the various States. It is of vital interest to the welfare of the profession that the question of reciprocity or inter-state comity should be settled so that, without any sacrifice of the very highest requirements, a physician in practice in one State having gone before a competent board, upon change of residence might be permitted to practice without being subjected to a second State examination, in the place of his adoption. The House of Delegates will, without doubt, act upon this matter at this session.

Referring to the subdivisions of the scientific work of the Association, Article V of the Constitution empowers the House of Delegates "to authorize new sections which may from time to time be organized, as the necessity for their existence arises." With increasing membership and the consequent larger attendance, it may be imperative in the future to create new sections, but this should be done only after careful consideration, and not until it is demonstrated that the material of high scientific value offered to the twelve sections now existing is more than can be utilized in the time allotted for the meetings.

The by-laws require every member to register in one of the sections, and it would be well to limit each reader to a single paper before the section chosen. The Association should insist that the officers of sections exercise a most rigid scrutiny of the papers referred to them. If we are to achieve our high purpose, if we wish to attract to our organization the great bulk of the better element of the medical profession, we must present through our sections papers which demonstrate not only the high scientific attainment of the author, but the undoubted value of the material presented. We are judged by our works, and if, at our meetings, and in the publication of our papers in *The Journal* which carries them to all parts of the earth, any unworthy material finds a place, it can but reflect discredit upon the Association.

Article VI. of the new Constitution says: "The House of Delegates shall have authority to provide for and to create such branch organizations as may be deemed essential to the promotion of the welfare of the medical profession."

For the present I would not advise the establishment of these subdivisions, but ultimately it may be found necessary to divide the States and territories according to population and geographical position into district branches where meetings may be held at the convenience of the States represented, and without interference with the annual session of the National Association.

Let us hope that the various tri-State societies and the sectional organizations, such as the Southern Surgical and Gynecological Association, and the equally successful Mississippi Valley Medical Association, and others of like character, attracted by the high and unselfish aims of this organization, may appreciate the vital necessity of a united profession and vote themselves into district branches of the American Medical Association. Truly, in such a union there would be strength so potent, and influence so far-reaching that we could safeguard, without doubt, the material interests of the profession, elevate still higher the standard of medical education, secure the enactment and enforcement of just and rigid medical laws, enlighten and direct public opinion in regard to the broad problems of State medicine, and demonstrate to the world the practical accomplishment of our science.

Article VII. of the new constitution, which deals with "Sessions and Meetings," refers the place and time for holding each annual session to the House of Delegates. They are also, under Article IX., empowered to appropriate funds for defraying the expenses of the annual meeting, as well as for enabling the standing committees to fulfill their respective duties.

I would recommend to the consideration of the Association the propriety of selecting in each of the geographical subdivisions of the United States, in which the sessions are successively held, some suitable location which has been found to be well adapted to the work of the organization, and to which we could return when the meeting is again to be held in that section of the country. As a scientific body intent upon fostering the growth and diffusion of medical knowledge, it is of vital importance to avoid in the selection of our place of meeting everything that could detract from the closest attention to the scientific program. The smaller cities, with ample hotel accommodations and halls conveniently located, have always yielded a larger attendance before the sections, and consequently a greater benefit to our members than the cities of larger size with their multitude of distractions. Moreover, it seems scarcely in accord with the dignity of this great body to require through its committee of arrangements that the physicians of the State and place selected for the convention should be held responsible for the expenses of that meeting. Every suggestion of commercialism should be avoided, and this prosperous organization should assume the entire responsibility and management of these annual sessions.

One of the most important duties imposed upon the House of Delegates is the selection of those who conduct its business affairs. In the past, the Association has shown a keen discernment in securing for its trustees and standing committees men not only of executive ability but held in high esteem as representatives of a profession which, according to the Code of Ethics, "should be temperate in all things, and which requires greater purity of character and a higher standard of moral excellence than any other calling."

You will in the regular order of business hear the reports of your five standing committees, and I am called upon to speak of but one, viz.: the Committee on Medical Legislation which the by-laws, adopted in June of 1901, directs to be appointed by the President, and to consist of one delegate from each State. In accordance with this requirement I mailed to the President of each State and territorial organization in affiliation with the American Medical Association a letter asking him to nominate one member for this committee. To all replies to this letter the name of the delegate was sent to the Chairman of the National Committee on Medical Legislation, Dr. H. L. E. Johnson, Washington, D. C. It will be remembered that at the session in St. Paul in 1901, the Association ruled that the National Committee on Medical Legislation, consisting of Dr. H. L. E. Johnson, Washington City; Dr. W. H. Welch, Baltimore, and Dr. W. L. Rodman, Philadelphia, should be continued until the meeting in June, 1903, and should have the same power to act in the interest of the Association that they had previously enjoyed. All the legislative affairs of the

Association I have referred to this committee at Washington, and have authorized them to call the full Committee on Medical Legislation for consultation, advice or aid whenever their services might be required.

In his message to Congress, December, 1901, the President recommended the establishment of a Department of Commerce and Industries. In its passage through the Senate the name was changed to that of Commerce and Labor. Before the national Legislature at the same time was a bill known as the Perkins-Hepburn bill to increase the efficiency and change the name of the United States Marine-Hospital Service to that of the United States Health Service, transferring this from the Treasury to the new department.

The American Medical Association has on several occasions expressed its desire for the establishment of a Department of Public Health, either as a separate department of the government, or as one of the important bureaus of a department. Probably on account of a lack of thorough organization and co-operation, it has not been able to obtain this important recognition for the medical profession. In view of these repeated failures it would seem advantageous to the scheme of establishing ultimately a Department of public health that the Perkins bill should become a law, because the United States Marine-Hospital Service could then with more propriety be removed from the new Department of Commerce and Labor, into a separate and independent department. This department should be in charge of a medical officer to direct our foreign and insular quarantine, interstate quarantine, the medical supervision of epidemics, and in fact all matters pertaining to the general health of any group of States or of the entire country.

The work of this officer and bureau can only be carried out with success by the earnest cooperation of the health officers of the various localities and States, and of the advisory board for the hygienic laboratory provided for in the Perkins-Hepburn bill, for the national and local authorities acting in harmony would be better able to prevent the importation of disease and to stamp out epidemics which may occur despite the greatest vigilance, and this with the minimum disturbance of the resident public and of the commercial interests of more remote sections.

As the representative organization of the medical profession of the United States, it is our duty to cooperate with the medical corps of the Army in the effort to procure legislation which will not only uphold the rights and dignity of the medical officers in the public service, but will give better protection to the health and lives of our troops.

The status of the Medical Department of the United States Army is fairly stated in a circular issued by the medical officers stationed in the Philippines, in which they claim that the present condition of affairs "is regarded as a menace to the efficiency of the medical department, as it is felt to be unfair and unjust." In no other staff department is promotion so slow as in the medical department. It is graded for rank, promotion and pay below every other staff department of the army, and, with the exception of second lieutenant, is graded below the line. A medical officer, under the provisions of the present law, to obtain a colonelcy must pass through three times as many files as an officer of the Quartermaster's, Subsistence or Pay departments; through more than twice as many files as an officer of the Engineers' or Ordnance departments, and more than one and one-half times as many as an officer of the signal corps. Officers of the line, having attained the rank of major, have to pass through but four files to obtain the rank of colonel, while the medical officers have to pass through nine files. All these facts are fully appreciated by the younger physicians of our country, and by the volunteer and contract medical officers, hundreds of whom are now serving with troops and are declining to become candidates for a position offering such an unpromising career and so little in the line of promotion and emolument.

The Secretary of War has been officially informed by the Surgeon-General that the number of available medical officers is being rapidly diminished, and he anticipates he will soon be unable to supply the demand for medical officers to replace those constantly returning from the Philippines, unless the prohibition placed by the Secretary of War upon the appointment of additional contract surgeons is removed. He says: "The service would no doubt be more attractive to well-educated physicians if the prospects for promotion were better, and I respectfully commend that Congress be asked at the present session to add to the medical corps of the Army, two colonels, six lieutenant-colonels and twenty-five majors. This would give thirty-three additional vacancies and would furnish an incentive to volunteer medical officers and contract surgeons now in service to seek admission to the regular army. I would also recommend that the age limit for volunteer surgeons and contract surgeons who have rendered satisfactory service for two years or more be raised to thirty-six years."

No one who has carefully studied the subject can but conclude that under the statutes now in force many lives have been sacrificed and much suffering has resulted from lack of thorough cooperation between the officer in command and his chief surgeon, and without doubt it would be to the interest of the service if medical officers were always consulted with regard to the location of camps and military posts for the purpose of getting expert opinion upon sanitary questions.

In order to impress upon commanding officers the importance of military hygiene and the greater necessity for this cooperation with the medical corps of the army, the Surgeon-General has insisted that there should be established at the Military Academy at West Point a course of instruction on military hygiene.

It is the duty of this Association to lend its best efforts to the Surgeon-General, a former president of the American Medical Association, and one not only in a position to suggest the legislation which would best serve the interests of the army, but one whom we know to be zealous of the interests, rights and dignity of the medical officers of the war department.

The committee to whom was entrusted the question of vivisection has been diligent, and it would seem successful, in its efforts to prevent unwise and injurious restrictions upon this important method of research. The wide dissemination of the contagion of smallpox in the United States within the last few years demands the most earnest attention of the medical profession. Such ignorance or indifference to the immunizing power of vaccination is a matter of surprise in an advanced stage of civilization, and, while laws for compulsory vaccination would without doubt be to the best interests of the whole people, it seems so contrary to the spirit of our institutions as to be impolitic as well as impracticable. It falls upon us as physicians to labor unceasingly to impress upon the communities in which we reside the necessity and safety of this immunizing process.

We will be wise if, from time to time, we make a critical analysis of our past, realize exactly what we are doing, and upon this, base such conduct as will assure to our successors a more satisfactory condition of our profession and a higher achievement of this Association. Being human we are too apt to shut our eyes to unpleasant truths; to exaggerate the value and the importance of our own performances, and to think that what we have been taught to believe, or what we wish to believe, is right and unchangeable. Let us ask ourselves plainly: Is the medical profession of the United States what it should be? Has it won the influential position to which it should aspire? Has it gained the power to secure just and proper legislation? Has it lived up to its obligations, and has the American Medical Association, which claims to represent one hundred and twenty thousand regular practitioners of medicine in the United States, fulfilled its mission. How many of us after due reflection can consistently answer these questions other than by saying plainly and regretfully—No. And wherein lies our weakness? To say we are part of a young and scarcely organized country; that our profession is widely dispersed over vast regions so remote from each other that contact and cooperation are difficult will not entirely satisfy the fairly critical mind. Such excuses might have been sufficient at an earlier date, but not now. To say that, despite these and other embarrassments, we of the United States have given to mankind the unequaled boon of ether anesthesia; that, through the achievements of members of our profession and of this Association, medical and surgical science has been greatly enriched; that great specialties recognized the world over have been developed; that operations bold and original have been established beyond controversy; and that by reason of these various contributions to the science and art of surgery and of medicine millions of lives have already been saved, together with the merciful mitigation of suffering which all this implies; while a repetition of this may flatter our vanity, it will not wholly satisfy us when in honest purpose we realize how great are our shortcomings.

It is a fact painful to acknowledge that of the three so-called learned professions—the ministry, law, and medicine—ours is accorded the inferior position, and we who day in and day out, in every home of the land, are close in the personal friendship of our patients, respected and loved as individuals, are incapable of wielding by organization and discipline the powerful influence of a united profession aiming at a high and honorable purpose. And what have been the results of this house divided against itself? Witness the snaillike progress which marked the various steps in securing our laws for elevating the standard of requirements in medical education and for medical practice. Witness the opposition to our efforts in securing better sanitary regulations, and in the struggle to protect the public from the horde of uneducated or misguided persons who, under the guise of Christian Science, Osteopathy, and other schisms, insist upon being permitted to take charge of and treat human beings suffering from disease without submitting themselves to the State examination legally required of us.

There are, in my opinion, two principal causes of evident weakness of the profession. First, the insufficient methods of medical education which have prevailed for the greater part of the first century of our national existence. Second, the lack of organization.

The Code of this Association says: "Those admitted into our ranks should found their expectations of practice upon the extent of their qualifications." We stand committed as the champion of higher medical education and the elevation of the standard of requirements applicable not only to the entrance examination, but to a rigid examination before the degree is received, as well as by the State before permission to practice is granted. To this rigid examination this Association, by its rules of conduct, demands another important essential. The highest order of learning, the greatest amount of skill may not make one an honor to our calling, for "there is no profession from the members of which greater purity of character and a higher standard of moral excellence are required than the medical, and to attain such eminence is a duty every physician owes alike to his patients and his profession."

The American Medical Association is the sponsor for organized medicine in the United States, and failure to accomplish this end implies the failure of this Association. We must not fail, nor will we, unless we falter in carrying out the plan of reorganization in the liberal and progressive spirit which characterized the session of 1901. It is our plain duty to endeavor to bring about the adoption by the various constituent bodies of a practically uniform constitution and by-laws for each county and for each State, modified only as the local conditions may require, and all governed by the same rules of conduct. These rules, as at present given in the Code of Ethics, adopted many years ago by this Association, should be also a subject of serious consideration at this time, for we cannot claim consistency or be logical in argument until there is but one code for the national Association and for all of the State organizations represented in the national body. This, as you well know, does not now prevail. Some years ago there lived and labored among us for the good of mankind and the honor of the profession a man whose genius was of the highest order and whose fame carried the name of American surgery throughout the world. He was one of those fearless pioneers in science who found his place ever on the frontier, clearing the way for those who were to follow. In 1876, at the meeting of the American Medical Association in Philadelphia, Dr. J. Marion Sims, in his presidential address, referring to the Code of Ethics, says: "The time will come when your organic laws, like the constitution of our country, will require modifications and amendments to suit a higher intelligence, a broader education and a greater destiny." In my opinion, the time has come when we cannot absolve ourselves from the responsibility of doing away with the inconsistencies for which we may now be properly criticized.

Such have been the changes in the statutes of a majority of the States since the Code was adopted by the respected founders of this Association, that we find it insisting upon conduct on the part of our members which is contrary to the laws of the States in which they reside. For instance, one section forbids a member of the regular profession to act upon a board of examiners which has to pass upon the legal qualifications of persons not graduates of regular medical colleges, while in thirty-eight of the States presented here, the civil statutes require these boards, which are composed in great part of members of the Association, to examine, pass upon and sign certificates or licenses to practice of Homeopaths, Eclectics, and other subdivisions of medical practice. In six of the States, including the District of Columbia, the law requires three separate examining boards. In Mississippi, North Carolina and South Carolina, the examining boards are entirely composed of regular physicians, and in one of these (Mississippi), while none but regulars are allowed on the board, the law explicitly says: "Distinction shall not be made between applicants because of the different systems or schools of practice that may be chosen." In almost all the States and territories regular physicians are compelled by the laws of the States in which they reside to disobey the injunctions of this section of the Code of Ethics.

A modification of this and other sections of the Code must be a part of the liberal plan of reorganization which we have essayed.

In conclusion, I ask this Association to stand for more than the healing art. To laber for the alleviation of suffering and for the restoration of health is a noble avocation, but to teach our fellows how to avoid disaster is a prouder privilege and a higher duty. We should be teachers of men. How better can we protect the public from disease in all its various forms and insidious processes than by perfecting in every county and in every community an organization which shall be ever watchful and insistent upon obedience to the laws relating to the public health.—*St. Louis Medical Review.*

---

### SOME FRACTURES IN CHILDREN AND THEIR TREATMENT.*

The treatment of fractures in children with but few exceptions, is essentially the same as in adults, necessitating knowledge of the

*Read at meeting of the South Branch of the Philadelphia County Medical Society, November 29, 1901, by L. J. Hammond, M.D., of Philadelphia, Surgeon to the Samaritan Hospital and Out Patients Department, Methodist Hospital.

anatomic relations of the parts, as well as correct recognition of the line in the solution of the osseous continuity. This deviation is, however, sufficient to justify some consideration. As examples of fractures to be specially considered, may be mentioned intra-uterine, congenital, rickety and spontaneous, together with the more common separation of the epiphyses and greenstick form of fracture.

The most frequent seats of fractures in children are of the humerus, forearm, clavicle, thigh and leg, while fracture of the maxillæ, scapula, sternum and pelvis are so rare that time need not be consumed in considering them, nor is fracture of the rib frequently met with in children except in cases of violent crush.

True epiphyseal separation, and by this is meant a distinct separation of the epiphyseal cartilage from the osseous end of the diaphysis, occurs only in infants and is extremely rare, while osteo-epiphyseal separation is, according to Beck and Schudder, frequently observed between the ages of fourteen and seventeen. Here the fracture is not limited to the cartilage, but passes into the diaphysis, and when of traumatic origin is most frequently found at the upper and lower ends of the humerus, the lower end of the radius, the upper and lower ends of the femur and the upper end of the tibia.

This form of fracture, or rather separation with fracture, cannot appear after the period of ossification, which according to Quain occurs in the humerus from the second to the twentieth year; in the radius during the same period, while in the femur from the ninth month to the first year. The age, however, at which ossification takes place varies somewhat and depends also upon constitutional conditions; for example, in dwarfs and rachitic subjects the cartilaginous condition may continue up until the fortieth year. The tendency of epiphyseal separations is to produce premature ossification of the limb, which may stunt growth.

In rickets, a condition less frequently met in this country than in Europe, the great brittleness of the osseous tissue makes fractures possible as the result of the smallest degree of violence, fragility in scurvy, infantile palsy of long standing, and Beck considers tuberculosis, especially of the knee, predisposing to fragility of the femur.

*Causes.*—The causes of fractures in children may be due, as in adults, to direct and indirect violence, as well as to muscular contraction; the latter cause, however, is seldom a factor. As an example of the rarity of fracture by muscular contraction in children,. I have neither observed myself, nor can I find in literature any case of fracture of the patella, os calcis or olecranon, three most vulnerable positions for fractures to occur from this cause.

Fractures are found simple and compound, as well as incomplete and impacted. Incomplete fractures are observed in children as a result of bending and partial separation, causing a so-called greenstick fracture which is due to the yielding of the convex cortical portion of the bone, while the concave stratum, which is more flexible, is but bent. This infraction, as it is called, is frequently found in rachitic children in the tibia, fibula and femur. This also furnishes the type of fracture *in utero* (intra-uterine), which, when thus occurring, is said to be due to irregular muscular contraction.

As to the regions most frequently the seat of fracture, it may be stated that the extremities, including the clavicle, represent seven-eights of all fractures occurring in children ; the upper extremities occurring about three times more frequently than the lower :

Forearm about 18 per cent. Of this number 20 per cent. occur at the lower end of the radius.

Humerus about 20 per cent. Of this number 20 per cent. are either condyloid, supracondyloid or epicondyloid.

Clavicle about 15 per cent. Of this number about 40 per cent. occur at or beyond the outer third.

The femur in our experience is much less frequently involved than any of the long bones of the upper extremities. This view is at variance with some statistics, and is caused in all probability by the fact that most of the cases that have come under my observation have been in dispensary work, consequently such fractures as those of the lower extremities requiring bed treatment have been fewer.

The cases of this character that I have seen have occurred either at the surgical neck, at the upper third of the femur or at the lower end.

More cases of vicious union of fractures of the surgical neck and upper third of the femur have come under my observation than of any other fracture in children, and it can be readily understood why this might occur; an unmanageable child would make it extremely difficult to retain perfect immobilization of the leg.

*Symptoms.*—Objective symptoms of fractures in children are the same as in corresponding fractures in adults, while the subjective symptoms are obviously at variance, and owing to the inability of children to define pain, also to their general unmanageable condition, it is important that every detail and every known means for acquiring knowledge of the actual condition be observed. The seat of greatest pain complained of is by no means always the seat of fracture, since it must be remembered that referred pain is a possibility.

Pain is less intense in children than in adults, due frequently to, first, lessened mobility of the fracture, even when complete, because of the greater thickness of the periosteum, which in a large percentage of cases does not permit of displacement of the fractured ends; second, because a mere separation of the epiphyses may cause but slight pain or displacement.

Deformity, displacement and crepitus are not always present; one or all may be absent. Again fractures of the greenstick type are often so slight that it is not until after the active formative process has taken place that excessive callus indicates the position. Fortunately, however, the infantile bone favors this rapid formative repair and almost invariably results in good union.

The infrequency with which deformities occur is due to the active reparative qualities in these bones; consequently but few cases of non-union are on record. There are, however, a few cases recorded of non-union of fractures *in utero.*

*Abnormal Mobility.*—This is present in all fractures, especially of large bones, except such varieties as infraction, impacted fractures or fissures; it may, however, be unreliable in short bones, and frequently in the ribs.

*Crepitus.*—If no abnormal mobility, it is absent, as well as in cases of wide separation either of the epiphyses from the diaphysis, or from interposition of soft tissues.

Functional disability depends upon the kind of fracture as well as the shape of the bone. Incomplete, impacted fractures or infracted bone will not wholly disable the part, ofttimes not even as much as muscular contusion; this is especially deceptive in children.

Deformities—these will depend upon the location, extent and duration after injury; therefore in fractures where there is absence of preternatural mobility and crepitus, with but slight, if any functional disability, reliance must be placed on ecchymosis, localized pain, inspection, palpation and effusion. This latter in children is much greater in fractures about the joints than in adults, though it is much earlier absorbed.

Ecchymosis, which is due, of course, to extravasation from rupture of the small vessels, can be looked for by the third day. Associated with it is usually some degree of inflammation. This is marked in proportion to the extent of the fracture, and is therefore most pronounced in the direct variety.

Localized pain is a fairly constant symptom, and in children is especially pronounced at night, or when the child undertakes to sleep, this characteristic symptom being due to muscular relaxation permitting movement of the fractured ends.

With these cardinal symptoms of fracture, if diagnosis still remains doubtful, and especially if the fracture is in the neighborhood of joints, and it be either infraction, impacted fracture or fissure, the Roentgen rays should at once make clear its precise character and location.

There is at present no necessity for remaining in doubt as to whether or not fractures exist, nor of the character of the fracture, because of this modern accession to our diagnostic acumen. Its use will further lessen the necessity for painful manipulation. If the case is seen for the first time, hours, or as it has happened to all of us, days after the fracture has occurred, and the swelling, ecchymosis and effusion are so great that manipulation cannot be satisfactorily practiced without, an anesthetic should be given provided the X-rays are not accessible.

Shortening, if present, will also be of value, and measurement should always be undertaken. Especially is this of some value in complete fractures of the extremities; its positive value, of course, is not to be expected because of the lack of constancy of the anatomic points.

*Duration of Treatment.*—On this point there seems to be some reasonable difference of opinion. There are those who claim that fractures of the clavicle, humerus and radial end of the forearm have satisfactory union within three weeks. This has not however, been my experience, and I deem it unsafe to allow fractures under my charge to have the immobilizing apparatus removed earlier than

Five weeks for fracture of the lower end of the radius.

Five weeks for fracture of the lower end of humerus.

Four weeks for fracture of the clavicle.

Six weeks for the upper third of femur.

Five weeks for fracture of lower end of femur.

Six weeks for fracture of the tibia and fibula.

Many have been led to early discard fixation apparatus, especially in fractures about the joints, because of the popular belief that anchylosis is the result of adhesions resulting from plastic synovitis caused by trauma. Since our better understanding of antisepsis and bacteriology, this is known not to be the cause, but that such stiffness of joints is due not to anchylosis from this plastic exudate, but to faulty apposition which may be due to interposition of soft tissues, or to subsequent displacement, excessive bone callus or improper early treatment.

The disturbing element in the process of repair in children, as in adults, is either local or constitutional. Delay in repair is, however, rare, and when it does occur is the result of either delayed callus, interposition of soft tissue or faulty apposition.

*Treatment.*—It is not the purpose of this paper to advocate anything unusual or heretofore unheard of in the treatment of fractures in children. The purpose of the paper is rather to bring before you for discussion, the most satisfactory method of dealing with those fractures not analogous to correspondiug fractures in the adult. Among these differences those that have especially proven instructive to me have been such fractures as occur at the surgical neck of the femur, upper third of the thigh, radial end of the forearm, the several fractures at the lower end of the humerus, the lower end of the tibia and fibula and clavicle.

### NECK OF THE FEMUR.

This is usually of the greenstick type and is very difficult to recognize without the aid of the X-rays. About as far as one can get from the history and examination, will be that there has been a fall; there will be some outward rotation of the leg and some limitation of motion; pain will be slight or may be absent.

With this meager information and a slight amount of displacement, and absence of the other cardinal symptoms of fractures, one is not to be censured severely if the condition be mistaken for and treated as contusion. This fracture has been frequently overlooked and treated simply as contusion, a most serious error, as the outcome will in a large percentage of cases lead to coxa-vera; therefore if any doubt exists, X-rays should always be brought into use to reassure.

The earliest possible treatment is necessary, and should consist in securing absolute rest in bed, on the back, with perfect immobilization of the hip, thigh and body. This can be satisfactorily secured by a long outside and a short inside sand bag.

If the child be very young, too young to be kept quiet by this means, then a plaster-of-Paris dressing should be put on from the ankle to the axilla, this dressing being retained for four weeks.

### FRACTURE OF THE THIGH.

This is usually the result of direct violence, is often of the incomplete variety and of the greenstick type. When complete, the symptoms are the same as in corresponding fractures in adults. When incomplete, and perfect reduction cannot be secured, it should be converted into complete, and maintained so by either a snugly fitting plaster-of-Paris spika splint, from the calf of the leg to the axilla, the limb being held at a right angle to the body, or if the child is at such an age that it can understand the importance of remaining quiet, a posterior angular splint may be used instead. Cabot's splint with coaptation splints may also be used. This latter means is a very satisfactory method in cases of very young children where it is practically impossible to keep any other form of dressing in position, and has some advantage over the plaster of Paris in that it is less weighty.

Whatever form of dressing be used, extension secured from the knee should be added.

After four weeks the child should be allowed to be about on crutches, with a plaster dressing, or many times nothing more is needed than firm bandaging of the leg. Care should be taken after the child is well, to prevent too early use of the limb, which will ward against dangers of bowing. There should be no shortening.

### FRACTURES OF THE LOWER EXTREMITY OF THE RADIUS.

Fractures at this seat are generally traumatic, due to a fall upon the hand. Those who have carefully studied this fracture, agree that in addition to the epiphyseal separation, that fracture extends also into the bone. Displacement is never so great as in adults.

After perfect coaptation of the fracture, which is best secured by forceful extension and slight adduction of the hand, pads are so placed as to hold in position after correction, the carpal extremity which is usually broken off transversely, the fragment being drawn backward and outward by the action of the extensors of the thumb and supinator longus; a pad is used also to correct faulty position of the ulna which is displaced so as to appear to extend toward the palm and inner side of the hand.

After this correction has been made, the parts should be immobilized either by plaster-of-Paris dressing, or a plain anterior and posterior splint, or by the more universally employed method, Bond's splint.

Whatever immobilizing method be employed, it is well to leave the fingers free, in order to do away with the stiffness that would occur if they are included in the immobilizing appliance.

### CONDYLOID, SUPRACONDYLOID AND EPICONDYLOID FRACTURES OF THE HUMERUS.

In my experience, the internal condyle has been the most frequent seat of fracture, though not a few of the supracondyloid variety have been seen. The latter is of the oblique variety and is recognized in addition to the usual signs, by displacement upward of the radius and ulna.

Treatment of all these varieties of fracture in this position, is practically the same, namely, reduction of the fracture by the usual means; in case of the supracondyloid variety by forceful extension associated with acute flexion of the arm, while in the simple condyloid variety, the arm is placed at an angle, with manipulation in position. The entire arm, from the wrist to the axilla, should be securely fixed by means of a snugly applied plaster-of-Paris dressing, or the same immobilizing action can be secured by either an internal or anterior angular splint, well padded and securely held by means of a roller bandage. My preference has always been for the plaster dressing as advocated by Prof. Dennis of New York.

### LOWER END OF TIBIA AND FIBULA.

Fracture here is generally the result of direct violence, as from a sudden twist while the foot is held firmly. It will be either an osteo-epiphyseal separation of the fibula or fracture of either or both of the malleoli. When the lower fifth alone is the seat of fracture, there will be abduction resulting from the action of the perineal muscles, and the astragalus will lie against the malleolus. Not infrequently the internal lateral ligament will be ruptured.

Treatment for this condition, as well as when associated with fracture of the malleoli, is first, to extend the foot and abduct it, seeing that it is securely held in this position until a perfectly fitting plaster-of-Paris dressing is applied and hardened. Dupuytren's splint may also be used. Pads must be placed above the malleolus to prevent its slipping out of position. In the use of Dupuytren's splint it is important after placing the splint in position to draw the foot to the splint by a bandage. Keep the dressing on for five weeks, when crutches should be used for three weeks longer.

### FRACTURE OF THE CLAVICLE.

In children it is due to falls upon the shoulder (indirect violence), and the greater number will be found at the outer third.

If the fracture is so near the acromian end as to be between the coraco-acromial ligament, displacement will be slight, if at all.

When nearer the center than this point, deformity will be distinct rotation of the outer fragment, sometimes to the extent of being at a right angle to the sternal end of the bone.

In addition to the usual signs, there will be some droppings of the shoulder. Measurement may show also shortening between the sternal and acromial attachment.

The object of the treatment is to draw the shoulder upward, backward and outward. For this purpose no better application can be used than the Sayre's dressing, combined with placing the arm in the Valpeau position.

Of the various means of securing perfect inmobilization of a fractured bone, there are none that I have found more satisfactory, especially in children, in all simple fractures, in all parts of the body with the exception of the clavicle, than a correctly applied plaster-of-Paris dressing. This method is advocated by Prof. Dennis, of New York, and I have for several years employed it to the exclusion of all other mechanical devices. I have no fear with its use, after the fracture is in proper position, that there will be need for inspection, that swelling will cause any undue amount of pain, or that it will be too loose after swelling subsides, the objection claimed by some to its use. On the contrary, there has been, in my experience, much less swelling when this form of dressing has been employed than there has been with a less firmly applied splint and roller bandage. Even should it occur that inspection be necessary, the simple procedure of dividing the plaster so that it may be sprung apart sufficiently wide to permit the fractured member to be removed and inspected, it can then be reapplied and held together by adhesive plaster.

In all cases after the immobilizing appliance has been removed, passive motion combined with the use of dry hot air should be employed for twenty-five minutes every second day, massage should also be employed and the child encouraged to use the member.—*Penn. Med. Journal.*

---

### TREATMENT OF SUMMER DIARRHEA OF INFANCY.

Dr. C. G. Kerley presented this paper. He said that as the physician had a poisoned child to treat the indications were clear.

Every case, no matter whether mild or severe, should receive an initial purge of castor oil or calomel, preferably the former, and regardless of the source of the milk it should be stopped at once. Milk is a good culture medium for bacteria, and hence there was nothing gained by diluting the milk or pasteurizing or sterilizing it. As a substitute diet one of carbohydrates was to be preferred, but as the children soon tire of this its taste should be varied by adding small quantities of beef juice, beef broth, chicken broth or mutton broth. However these must be used very sparingly, as in some children they tend to produce diarrhea. It was a mistake to make free use of brandy or whiskey in these cases, for these still further weakened the already enfeebled digestive organs and irritated the kidneys. He had tried egg-albumen water, but had given it up because it was often not well digested and recovery was retarded by its use. The substitute carbohydrate diet should be given every two hours and in the same quantity as milk had been previously given. Boiled water might be given freely at any time. It was important to keep the sick child in a large and airy room and not to resume the milk till the fever had gone and the stools had become normal, and then to return to milk diet very gradually and cautiously. He had found the subnitrate of bismuth useful, but unless given in doses of ten to twenty grains every hour or two, in other words, in sufficient quantity to cause black stools, it had but little effect. Opium should be used with great caution and never unless there were more than four or five stools a day, as this numher was needed for intestinal drainage. The best form was Dover's powder, of which $\frac{1}{4}$ to $\frac{1}{2}$ grain should be given every three or four hours to a child one year old. Where a heart stimulant was indicated he preferred to use strychnin and strophanthus. If the stools contained much blood and mucus, irrigation of the colon would be found useful; otherwise it only served to increase the rectal irritation. Normal salt solution should be used for the irrigation and it should be administered through an ordinary rectal tube of soft rubber (No. 17 American scale), the reservoir for the solution being not more than three feet above the body of the child. For ordinary purposes the solution should be lukewarm, but if there were much fever this could be reduced by using the irrigation at a temperature of 70° F. On the other hand where collapse was imminent, the saline solution should have a temperature of about 110° F.

Dr. W. L. Stowell said that he had never found it necessary to pay any special attention to renal complications occurring in connection with infantile summer diarrhea. The statistics for the past three years at the Demilt Dispensary showed that summer diarrhea was on the wane and that the cases that did occur were of a milder type than in previous years. He was accustomed to prescribe intestinal antiseptics, such as salol, and to use rectal irrigation, not only for the purpose of removing irritating matter but because of the constitutional effect that could be produced in this way as hinted at in the closing remarks made by Dr. Kerley. Drugs played a very subordinate part.

Dr. B. Van Doren Hedges, of Plainfield, N. J., spoke of the baneful results of chronic distention of the stomach by giving children food in inordinate quantities. The treatment advocated by Dr. Kerley was most sound, but personally he was very chary of giving opium to children unless they could be very carefully watched. In no case should opium be kept up if along with the diminished number of stools there was no improvement in their character. This could only be determined by a personal inspection of the stools, and this was an important part of the physician's duty.

Dr. W. L. Carr said that in some cases presenting renal complications his attention had been attracted by the presence of vomiting, a very rapid pulse and dyspnea. Edema was not usually observed except in protracted or intermittent cases. The albuminuria was very slight, and indeed, the general mildness of the kidney complication explained the frequency with which it was overlooked. He had practically abandoned the internal administration of so-called intestinal antiseptics, and believed the children did just as well if treated according to the plan laid down in Dr. Kerley's paper.

Dr. L. E. Lafetra spoke of a rare case in which there had been a local instead of a general intestinal intoxication. This had apparently been brought about by paralysis of the intestine and the case had terminated fatally.

Dr. E. Libman's experience with the kidney complications had been very similar to that of Dr. Morse. He had observed usually a parenchymatous degeneration of the kidneys. Probably such cases were more marked in the old days when the treatment by opium and astringents held sway, because under such medication the elimination of toxins was materially interfered with. Dr. Libman made a plea for an attempt at least at a scientific classification of the infantile diarrheas. Certainly, he said, there was no difficulty in separating two classes—the streptococcus and the pyocyaneus cases, and this should be done even if such a classification did not in any way affect the treatment.

Dr. J. F. Bell, of Englewood, N. J., described an epidemic of infantile diarrhea that he had witnessed in Easthampton, L. I., during the summers of 1899 and 1900. The cases were very severe and were characterized by high fever, convulsions, vomiting and a high mortality. Bacteriological examination of the stools revealed the presence of streptococci, and as there was an unusual number of relapses on resuming milk diet, attention had been directed to the milk supply. It was found that many of the cows supplying milk to these children had an eruption on the teats and udders, and a culture from one of the pustules showed staphylococci and streptococci. Dr. E. F. Brush had been asked to see these cows and had pronounced the disease cowpox. There had been an almost immediate cessation of the epidemic on quarantining the diseased cows and procuring milk from healthy cows. A similar outbreak had occurred in the neighboring manufacturing town of Sag Harbor.

Dr. W. L. Baner spoke in terms of high commendation of the treatment as outlined by Dr. Kerley. His own preference was for sulphate of sodium rather than bismuth, and he prescribed it in doses of fifteen grains every three or four hours to a child a year old.

Dr. J. J. Walsh decried the prevalent notion that salol and various coal-tar preparations supposed to inhibit the activity of the germs in the intestine must be prescribed in these cases. An excellent prophylactic against summer diarrhea during very hot weather was the frequent bathing of children or allowing them to play in a bath for a considerable time.

Dr. Morse suggested that summer diarrheas, or what he preferred to call the acute enteric diseases of infancy, be roughly classified as follows: (1) A class in which there was merely simple diarrhea or increased peristalsis; (2) a class in which there were organic changes; and (3) a class in which there might or might not be organic changes. The third class included the great bulk of all cases. While milk should certainly be prohibited for a short time, he believed it could be resumed sooner than Dr. Kerley seemed to think. Brandy and whiskey were contraindicated and opium should only be used where there was undue peristalsis. In the latter class such astringents as tannigen and tannalbin were also very useful. Rectal irrigation had been very much overdone; it was applicable to a comparatively small number of cases. The subcutaneous injection of saline solution was most useful in the very severe forms of infantile summer diarrhea.

Dr. Kerley remarked that as salol was not beneficial, and almost always disturbed the stomach, it should be avoided.

Dr. R. G. Freeman said that the physicians working in the New York Foundling Hospital had never been greatly impressed with the value of bismuth in these diarrheas under discussion, but since they had been making use of bismuth associated with two or three drops of castor oil they had obtained satisfactory results from this combination.—*Pediatrics.*

---

### A CONTRIBUTION TO THE THERAPEUTICS OF ANEMIC CONDITIONS.

BY DR. HERMANN METALL,
Assistant Physician to the General Polyclinic, Vienna.

(Translated from the German.)

In the medical treatment of the various forms of anemia, whether it be essential chlorosis or the so-called secondary forms arising from severe loss of blood and various diseases (tuberculosis, cancer, etc.), iron has always occupied the most prominent place. In the management of chlorosis, especially, the chief object is the administration of an adequate quantity of iron, since upon this depends the success of all treatment. As to the manner in which iron acts in anemic conditions, that is a secondary matter. Whatever be its mode of action, it remains an empirical remedy and yet one of incontestable value.

According to the unanimous opinion of many authors the effect of iron in chlorosis cannot be replaced by alimentation. Reinert, Klein, Immermann, Ensli, and others have shown that typical chlorosis cannot be cured in any other way, even by forced feeding. Some of them have made a series of very careful experiments for this purpose, and reached the remarkable result that during superalimentation, extending even over a number of weeks, the quantity of hemoglobin in the blood increased scarcely a few per cent., and remained permanently at this level. That this is actually so we daily convince ourselves in cases of chlorosis in girls of the better classes. These girls, if placed on a full diet, accumulate more fat, while the chlorosis remains practically unaffected—it requires iron. The dietary therefore plays a subordinary part in the therapy of chlorosis (Klein), and is to be regarded only as an important adjunct to the treatment.

I will now devote a few words to manganese, which is employed in combination with iron in some ferruginous preparations for the treatment of anemia. Hannon already directed attention to this metal, which is a constituent of healthy blood, and which besides iron has an important bearing on the absorption of oxygen by the blood. In fact, experiments have shown that anemic conditions are most successfully treated with iron in connection with manganese. Chalybeate medication is materially aided and promoted by the addition of manganese. Efforts have therefore been made to introduce combinations of iron and manganese into therapeutics.

After laborious attempts, Dr. Gude, chemist, succeeded in producing such an iron-manganese preparation, which is easily absorbed by the entire intestinal tract, evokes no concomitant effects, and, as is illustrated in the following histories of cases, has proved an excellent remedy for the formation of blood. The preparation referred to is Pepto-Mangan (Gude). It contains iron and manganese in an organic combination with peptone, and is a clear fluid, resembling dark red wine, of an agreeable, non-metallic, non-astringent taste.

The advantage of this preparation is that it exerts a stimulating effect upon the blood-forming organs, these being excited to greater functional activity, and that the favorable effect manifests itself even within a short time by an increased oxygenation of the blood. At the same time, this chalybeate, as already mentioned, causes no digestive disturbances and does not injure the teeth.

In regard to the daily dose of iron, Quincke maintains that it should range from ¾ to 1½ grains of Fe. Most clinicians prescribe commonly 4 grains, which considerably exceeds the maximum dose recommended by Quincke. Some of them, like Niemayer and Trousseau, give even 7 grains of metallic iron daily; hence Pepto-Mangan (Gude) should be prescribed in doses of one tablespoonful three daily for adults, and teaspoonful twice daily for children up to twelve years, after meals. Sour, fatty foods and red wine should be avoided during its administration. The preparation is much relished by all patients, and it is my custom to administer it to children in water, or, better, in cold milk with the addition of sugar, in which form it is very palatable.

After this brief introduction I will describe a number of cases which have been treated by me with Pepto-Mangan:

CASE I.—Mary B., 16 years old, has complained since a week of general debility and lassitude. She is very pale and restless, has no appetite, and suffers from headache and a feeling of pressure in the stomach. She is constipated, and the menses are irregular. Diagnosis, chlorosis.

| Date. | Red Blood Cells in Cubic Millimeter. | Hemoglobin per cent. | Bodily Weight. | Therapy. |
|---|---|---|---|---|
| August 2........ | 2,480,000 | 20 | 49.2 | Pepto-Mangan (Gude), one tablespoonful three times daily. |
| August 9........ | 3,212,000 | 25 | 50. | |
| August 16....... | 4,020,000 | 30 | 50.5 | |
| August 24....... | 4,300,000 | 40 | 51.3 | |
| September 2.... | 5,000,000 | 50 | 53.4 | |

After a week, the appetite was good, no headache; at the end of the second week, no further disturbances; menses not painful, and lasting only three days (formerly five days). After four weeks, the patient discharged cured.

CASE II.—Anna H., 23 years old, has suffered for three years from chlorosis, with irregular menstruation, palpitation of the heart, a feeling of weakness, and occasional syncope. Physical examination showed the presence of anemic murmurs over the heart, as well as a venous murmur; no fever or edema.

| Date. | Red Blood Cells in Cubic Millimeter. | Hemoglobin per cent. | Bodily Weight. | Therapy. |
|---|---|---|---|---|
| August 4........ | 3,750,000 | 35 | 55.5 | Pepto-Mangan (Gude), one table-spoonful three times daily. |
| August 29....... | 4,010,000 | 60 | 57.8 | |
| September 14... | 4,200,000 | 70 | 59. | |

Appearance of menses after absence of 12 weeks; subjective disturbances have disappeared.

CASE III.—M. W., 15 years old, has suffered since a year from headaches, dyspnea, tinnitus aurium, vertigo, and gastric disturbances. There was marked pallor of the face and of the mucous membranes; systolic murmurs over the mitral and pulmonary valves, with dilatation of the heart. No fever; spleen not palpable. Diagnosis, severe chlorosis.

| Date. | Red Blood Cells in Cubic Millimeter. | Hemoglobin per cent. | Bodily Weight. | Therapy. |
|---|---|---|---|---|
| August 5........ | 2,250,000 | 25 | 52.5 | Pepto-Mangan (Gude), one table-spoonful three times daily. |
| August 13....... | 3,200,000 | 30 | 53.5 | |
| August 16....... | 3,350,000 | 35 | 55.5 | |
| August 23...... | 3,530,000 | 40 | 56.5 | |
| September 1.... | 4,250,000 | 45 | 58. | |

The subjective symptoms rapidly subsided, the appetite improved, and the stools became regular. The menses reappeared in the second week of treatment after having been absent for a year.

CASE IV.—M. P., 15 years old. Menses absent since one-half year; always scanty. Vicarious hemorrhages from the nose. Since three months the patient has suffered from dyspnea, vomiting, cardiac palpitation, general weakness, headaches, feeling of dullness, and sleeplessness. Physical examination reveals anemic murmurs, moderate dilatation of the heart, venous murmur.

| Date. | Red Blood Cells in Cubic Millimeter. | Hemoglobin per cent. | Bodily Weight. | Therapy. |
|---|---|---|---|---|
| August 5........ | 2,400,000 | 20 | 47. | Pepto-Mangan (Gude), one table-spoonful three times daily. |
| August 10........ | 3,600,000 | 25 | 47.5 | |
| August 16........ | 3,850,000 | 30 | 48.5 | |
| August 23........ | 4,250,000 | 35 | 49.0 | |
| August 31........ | 4,700,000 | 40 | 49.7 | |
| September 7.... | 5,000,000 | 45 | 52. | |
| September 14... | 5,200,000 | 50 | 53. | |

After the first week improvement set in; at the end of treatment disappearances of all disturbances. Increase of bodily weight, 12 pounds.

CASE V.—J. K., 18 years old. Chlorosis. Anemic murmurs, cardiac dilatation, loss of appetite, insomnia, general lassitude, and headaches.

| Date. | Red Blood Cells in Cubic Millimeter. | Hemoglobin per cent. | Bodily Weight. | Therapy. |
|---|---|---|---|---|
| August 10........ | 2,200,000 | 35 | 52. | Pepto-Mangan (Gude), one table-spoonful three times daily. |
| August 24........ | 3,000,000 | 45 | 55. | |
| September 12... | 3,300,000 | 60 | 57. | |

At the end of the first week, appetite vigorous; headaches had subsided. At the end of the fourth week no disturbance of any kind.

CASE VI.—A. N., 19 years old, has suffered from chlorotic disorders since two years. Improvement occurred under a milk diet and a sojourn in the country. Since five months the patient again complains of disturbances: palpitation of the heart, lassitude, headache, vertigo, tinnitus, and constipation; anemic murmurs and venous hum perceptible.

| Date. | Red Blood Cells in Cubic Millimeter. | Hemoglobin per cent. | Bodily Weight. | Therapy. |
|---|---|---|---|---|
| August 17........ | 4,500,000 | 25 | 53.5 | Pepto-Mangan (Gude), one table-spoonful three times daily. |
| August 25........ | 4,100,000 | 30 | 54. | |
| August 31........ | 4,000,000 | 35 | 54.5 | |
| September 7.... | 3,950,000 | 40 | 56. | |
| September 22... | 4,200,000 | 45 | 57.5 | |

The subjective symptoms diminished after a few days. The disturbances disappeared, the appetite improved, and the stools became regular.

CASE VII.—J. R., 20 years old, has suffered from chlorosis since two years. Status præsens: General lassitude, palpitation of the heart, a feeling of pressure in the stomach, difficulty in breathing; menses irregular as well as dysmenorrhea. In the last three months all the disturbances have become more intense.

| Date. | Red Blood Cells in Cubic Millimeter. | Hemoglobin per cent. | Bodily Weight. | Therapy. |
|---|---|---|---|---|
| August 22. ..... | 4,250,000 | 30 | 52. | |
| August 26....... | 4,350,000 | 35 | 52.5 | Pepto-Mangan |
| September 5.... | 5,420,000 | 40 | 53.5 | (Gude), one table- |
| September 12... | 5,300,000 | 50 | 54. | spoonful three |
| September 18... | 5,350,000 | 55 | 54.5 | times daily. |
| September 27. . | 5,300,000 | 60 | 55.5 | |

The disorders have disappeared, the appetite is good, and the bowels regular; no anemic heart murmurs.

CASE VIII.—L. N., 19 years old, complains of headaches, cardiac palpitation, vertigo; scanty menses.

| Date. | Red Blood Cells in Cubic Millimeter. | Hemoglobin per cent. | Bodily Weight. | Therapy. |
|---|---|---|---|---|
| August 22... ... | 2,500,000 | 40 | 54. | Pepto-Mangan (Gude), one table- |
| September 13... | 3,750,000 | 55 | 55.5 | spoonful three |
| October 1....... | 4,300,000 | 70 | 57. | times daily. |

The subjective disorders have vanished; menses more abundant.

CASE IX.—J. M., 16 years old, has suffered since two months from palpitation of the heart, dyspnea, feeling of pressure in the stomach, vertigo, tinnitus, and headaches. There is a slight cardiac palpitation, with systolic murmurs and a venous hum. Anorexia and constipation are present. The menses have been irregular for a year.

| Date. | Red Blood Cells in Cubic Millimeter. | Hemoglobin per cent. | Bodily Weight. | Therapy. |
|---|---|---|---|---|
| September 2.... | 4,500,000 | 35 | 50. | Pepto-Mangan |
| September 11... | 4,750,000 | 40 | 50.5 | (Gude), one table- |
| September 20... | 4,850,000 | 50 | 51. | spoonful three |
| September 29... | 4,950,000 | 55 | 52. | times daily. |

Menses regular; bowels normal; no disturbances.

CASE X.—Z. F., 30 years old, had a miscarriage two weeks previously, with profuse hemorrhage. After a month's treatment completely restored to health, and an increase of weight of four pounds.

CASE XI.—A. N., 6 years old; rachitis and anemia. Under treatment an increse of weight of two-thirds of a pound. Much better appearance.

CASE XII.—J. W., 30 years old. Pulmonary tuberculosis and anemia: After two weeks' administration of Pepto-Mangan (Gude), an increase in weight of two pounds and an increase in hemoglobin of *fifteen* per cent.

CASE XIII.—K. L., 50 years old. Cancer of the stomach, cachexia and anemia. During three weeks' use of Pepto-Mangan (Gude) the patient felt better, the appetite had improved, and there was an increase of weight of two-thirds of a pound.

CASE XIV.—A. B., 14 years old. Chlorosis; hemoglobin 40 per cent. After two weeks' treatment, hemoglobin 85 per cent.; disappearance of all disturbances.

CASE XV.—F. K., 18 years old. Chlorosis; hemoglobin 35 per cent.; after two weeks' treatment 50 per cent.

CASE XVI.—E. J., 5 years old. Anemia following scarlatina. After eight days' treatment with Pepto-Mangan (Gude) the patient developed a vigorous appetite, and recovered so rapidly that he could be discharged cured at the end of the second week.

Altogether, twenty-three cases of anemia were treated with Pepto-Mangan (Gude), of which twelve showed a normal hemoglobin per cent. of the blood after fourteen days, five after three weeks, and five after a month. On the other hand, one of the patients who had hereditary trouble (her father having suffered from pulmonary disease) was discharged only improved, the blood, after two months' treatment with Pepto-Mangan (Gude), showing only an increase of hemoglobin to 75 per cent. This was probably a case of tuberculosis which simulated an obstinate or severe chlorosis at its beginning.

Furthermore, two cases of acute anemia after profuse hemorrhages were treated with Pepto-Mangan (Gude). A favorable result was obtained as early as the end of the first week. In one instance the patient felt so well that only the fear of further hemorrhage constrained him to stay in bed for another week. In the case of three women who had miscarried during the early months of pregnancy, and were making a very slow recovery from the resulting anemia, I was able to obtain a complete recovery after four weeks' administration of Pepto-Mangan (Gude). In six other instances of weakness and anemia following acute and chronic disease (tubercolosis, carcinoma, scarlet fever, etc.), a disappearance of the feeling of weakness and a considerable improvement of the general health could be observed in every instance.

The histories cited above will afford conclusive evidence of the high therapeutic value of Pepto-Mangan (Gude). Unpleasant concomitant effects and disagreeable sequelæ were *never* observed during the use of the remedy. Eructations, pressure in the stomach, and nausea were never noticed.

In conclusion I would say that Pepto-Mangan (Gude) is a valuable and reliable blood-building remedy, which can be recommended for general use in appropriate cases.

---

### ALCOHOLISM AND CRIME—HOW WE SHOULD DEAL WITH THE CRIMINAL ALCOHOLIC.*

Judges of criminals, prison wardens, and the police unanimously concede that at least seventy per cent. of all perpetrated crimes are directly or indirectly attributable to alcoholism. This unusually large percentage applies principally to such offenses as the disregard of the rights of others, and contempt for law and order, and to such crimes as assault, rape, disturbance of domestic

*Heinrich Stern, M.D., of New York City, in *American Medicine*, Feb. 1, 1902.

peace, manslaughter and robbery, for to all of these the habitual drunkard seems to be particularly prone. Such misdemeanors are frequently committed in a moment of passion, hence entirely unpremeditated, and so soon as the perpetrator regains his normal senses he is seized with remorse. While the sober man, it is true, is also subject to sudden and violent emotions, he is, nevertheless, by exercise of his will-power, able to curb and control the impulsive and irrational dictates of his passions. He is well aware of the consequences awaiting him upon the perpetration of a crime, and weighing them in his mind, will either abandon his project or exert his utmost precaution while executing it. While the sober man is master of his impulses, the drunkard is a slave to his emotions and passions. At the slightest provocation the inebriate may commit an assault or even manslaughter, and if an opportunity offers itself, he may commit moral delinquencies, as rape, etc. But he rarely commits such offenses as are the result of premeditation or design. Of all those convicted of perjury, but twenty-six per cent. or twenty-seven per cent. are addicted to alcohol, for perjury is based on some subtle motive—either love of money or the endeavor to save another from punishment—in fact, motives originating only in the unclouded mind of the sober. Furthermore, amongst incendiaries we find but forty-five per cent. are alcoholics, for arson is premeditated and usually executed with a distinct purpose in view.

Alcohol habitually used affects principally the brain and nervous system, which lose their normal degree of resistance; their vitality and healthy activity are reduced, and a general deterioration of brain and nerve substance is the inevitable result. More remote consequences are generally retrogression of intellect, debased standard of morality, and complete or partial loss of will-power. We need not descend to the confirmed drunkard to find evidence of general deterioration. Even in the milder form of habitual drunkenness we find similar conditions. The habitual alcoholic shows signs of premature deterioration; his mind is more or less inactive, his general bearing is undignified, his sentiments are more vulgar and his sense of truthfulness is deficient. His standard of morals is lowered, his love of family gradually declines, and an indifference as to the future of his offspring becomes apparent. Furthermore, he develops ruder and brutish affections, becomes irritable, jealous and peevish, and obstinately adheres to his preconceived irrational ideas and notions. The "bonhomie" and congeniality of certain drinkers is virtually but a species of indifference, but one of the protean manifestations of chronic alcoholism.

Of all the pernicious effects of alcoholism, none is so deplorable as the fact that the offspring must suffer for the cravings of its parent. The degeneration of children of drunkards is a fact which is still more apparent when both parents have been habitual alcoholists. The chronic alcoholic affection of the brain is more or less a disease of character. The character of the drunkard is abnormal, his standard of morals is lowered, and he conceives an entirely new moral code incomprehensible to the sober. The habitual alcoholic, like the morphine habitué, and like certain types of the insane, lives in a world of his own. He has his delusions and hallucinations, and while subject to them he is unable to distinguish between right and wrong. The chronic alcoholist is a psychopath, who ought to be adjudged and treated accordingly. The heavy drinker, bereft of any moral sense, is without any ambition or energy. He is a coward at heart, but under the influence of alcohol he may vacuously commit a daring act of the consequences of which, however, he has no clear conception. He cannot resist temptation, and in the association with criminals he soon becomes a criminal himself. His will-power, self-determination, and clear judgment being undermined, he is often a willing tool for their adventures and culpable enterprises. Hence, association with low characters is another cause of the further downfall of drunkards.

According to our present laws, the habitual or periodic drunkard who has committed a slight offense is imprisoned in a penitentiary or a workhouse, where he is in close proximity and contact with criminals. Necessarily, this must be detrimental to him who is so little capable of exerting self-determination. The alcoholist, who was perhaps but an accidental offender, may thus become an incorrigible criminal. Prison statistics prove that the more frequently an alcoholist is incarcerated, the more incapable of reformation he becomes.

If the purpose of punishment is prevention of crime, reformation of criminals and the protection of society, then the prison is the wrong place for the inebriate offender. There the alcoholist is rarely cured of his disease; there he develops a still stronger craving for liquors, and from there, in the great majority of instances, he graduates a full-fledged criminal, a fact of which every police officer of experience, philanthropic institutions, and magistrates are well aware. But in spite of the disastrous condition confronting them, they have not devised any means to successfully cope with it. As the main object of sanitary science and modern medicine is prophylaxis of disease, so prevention of crime should be the endeavor of the sociologist and legislator. Agreed, if we are convinced that the drunkard is prone to commit crime, there is no reason why he should not be confined before he has occasion to do wrong. There ought to be attached to every police court an experienced medical officer, whose duty it would be to inquire into the antecedents pertaining to the physical and mental condition of the accused, and to determine whether he is suffering from the consequences of an alcoholic toxicosis. This officer should classify the different types of inebriates at least into two great groups: (*a*) the occasional offenders, and (*b*) the confirmed alcoholists. The result of his examination should be taken into due account by the police magistrate, who should sentence both the first offender and the "rounder" to confinement in public reformatories devoted to their detention, and to the rational treatment of drunkards. The two classes would be kept separately in these institutions, or what would be still better, separate institutions should be provided for the treatment of culprits of each of these groups.

The asylum or institution, which should be managed by a special Honorary State Board, or by a Commissioner, should be established in farming districts, so that the convicted drunkard may engage in agricultural pursuits, which, in the great majority of instances, are best suited to his peculiar mental and bodily conditions. An institution of that character, conducted on a liberal and scientific basis, where moral, dietetic and medicinal treatment go hand in hand, where the convict is put to such manual labor as his constitution demands and permits, where rest and recreation are prescribed as the individual case exacts—such an institution will produce far superior results than may be obtained in general penitentiaries and behind prison bars. At least a part of the revenue derived from the liquor tax should be appropriated to defray the expenses of these penal institutions. To what better advantage and purpose could this revenue be devoted than for the amelioration of the spiritual and bodily condition of these individuals, who, by their incessant consumption of alcoholic beverages in public places, are almost the main source of the liquor tax income? The establishment of asylums for the criminal alcoholist would cause the transfer of at least half the inmates from the penitentiaries. A comparatively large sum, therefore, which is annually expended for the maintenance of the latter, could then be devoted to the support of these institutions. Furthermore, some penitentiaries, particularly those in rural districts, could be transformed into asylums for the criminal alcoholists, such changes involving little labor or expense. With some good-will, and with the assistance of the medical and legal professions, the object in view may be readily called into existence, so much the more so as it is not a question of funds, but only one of system.

The criminal alcoholist who has spent his penal servitude—which should continue until he is cured, or if incurable, indefinitely—in an institution of such a character, leaves it regenerated. With the newly-acquired or regained energy and vigor, the former alcoholist is well equipped to start afresh in life—a healthy man, alive to the interests of home, community and country, and a useful member of that society which to-day condemns him and considers him an outcast.—*St. Louis Med. and Surg. Journal.*

---

### THE INSTRUMENTAL RELIEF OF ACUTE RETENTION FROM PROSTATIC ENLARGEMENT.

BY WILLIAM T. BELFIELD, M.D., CHICAGO, ILL.,
Associate Professor of Surgery in Rush Medical College.

The introduction of a catheter for the relief of acute retention from prostatic enlargement is, when intelligently done, entirely free from difficulty; but as frequently performed it becomes most formidable for both physician and patient. Hours of fruitless effort, lacerations of the urethra, serious hemorrhages, immediate suffering, and remote suppuration in the pelvic tissues, are common features of these cases. These unfortunate experiences, disastrous alike to the patient's welfare and the physician's reputation, are (as a rule) entirely unnecessary; they occur because the physician has an erroneous idea of the character of obstruction to the outflow, and therefore uses a metallic catheter.

It seems to be the general impression that the obstacle of urination in these cases is some rigid tissue, similar to an organic stricture; hence that some firm—i. e., metallic—instrument must be used to force a passage. A second dangerous deduction from the false premises is that this metallic instrument should be of small caliber. When called to such a case, therefore, the physician is prone to select a small metal catheter; this is passed into the penis and proceeds without obstacle until the beak enters the perineum. Here the instrument often meets an obstruction (perhaps the narrow opening into the triangular ligament); the physician, believing this to be the obstacle to urination, begins to force the instrument, gently at first, and more firmly afterward. After a few minutes of ineffectual pressure, the catheter is withdrawn somewhat for a fresh trial—and blood flows from the meatus, marking the first false passage. Thus the sanguinary contest with an imaginary enemy is begun.

The fact is that the obstacle to the exit of urine is not a stricture or other firm encroachment upon the urethral canal, but solely an edema of the prostate which offers but slight opposition to the entrance of a catheter, although it prevents the opening of the vesical outlet from within, and thus causes retention. Hence a flexible gum or silk catheter easily overcomes the obstruction.

But the serious objection to the use of the metallic catheter is this: Its curve is adapted solely to the passage of the normal urethra, while the urethra traversing the enlarged prostate is abnormal—distorted by the irregular hypertrophied gland. To attempt to pass a rigid instrument with a given curvature through a rigid prostatic urethra with an unknown but probably different curvature is evidently irrational and obviously invites the disaster that so often ensues—laceration of the prostate and failure to find the vesical orifice.

The proper instrument for introduction through a distorted, edematous prostatic urethra of unknown curvature is evidently one sufficiently flexible to follow the unnatural curves without laceration, and yet sufficiently rigid to transmit a certain slight force from the hand of the operator to the beak. A soft-rubber (Nélaton) catheter sometimes suffices, but often fails because too flexible and soft to separate the edematous walls of the prostatic urethra. The Mercier catheter—also called "prostatic" and "coude"—most intelligently devised by the noted French surgeon to meet this emergency, may be relied upon to relieve every case of acute retention from prostatic enlargement, unless the urethra has been lacerated by metallic sounds prior to its use. The stiffened, turned-up beak of this instrument hugs the roof of the urethra; as the prostatic tumors (as well as false passages) are usually found on the floor of this canal, it is evident that the Mercier instrument will evade both pitfalls as no other instrument can. Its shaft is sufficiently rigid to transmit the necessary force, yet so flexible that laceration of the urethra is impossible. (The physician should remember to mark the heel of the Mercier so as to identify the direction of the beak after the latter disappears from sight.)

The only cause for failure to introduce the Mercier catheter (aside from tight organic strictures, which are seldom met in these cases) is the presence of false passages; and even these may be often evaded by a Mercier of large caliber (15 English or more) when the small ones fail. But if the false passages be large and numerous, even the larger Mercier instruments may persistently traverse them instead of the natural channel. In this contingency—and in no other—a metal catheter should be tried; it should be of large caliber, long curvature, and its beak should be controlled and guided by the finger in the rectum. Under no circumstances should it be forced after its beak enters the prostatic urethra; for an obstacle requiring force to overcome it lies outside the urethral lumen, and should be avoided, not attacked.

If no instrument can be made to enter the bladder through the natural channel, an artificial exit must be secured above the pubes.

The simplest measure—aspiration—secures relief only for a few hours, and sometimes needs frequent and inconvenient repetition; hence if it become necessary to make a suprapubic puncture, this should be done with a small trocar and cannula. Through the latter a small Nélaton catheter is passed deeply into the bladder; the cannula is then withdrawn, and the catheter fixed in position by adhesive straps and safety-pins. The continuous drainage of the bladder thus afforded can be maintained until voluntary urination or introduction of a catheter is accomplished.

SUMMARY.

1. Acute retention from prostatic enlargement is due to edema—not a rigid obstacle—in the prostate.

2. This edema can be passed by a Nélaton catheter, usually; always by a Mercier catheter.

3. A metallic catheter being curved to traverse a normal urethra is not adapted to the elongated, distorted, congested canal of prostatic enlargement; hence must never be used except as a last resort; and then with utmost care.—*American Journal of Surgery and Gynecology.*

---

### SARATOGA MEETING OF THE ASSOCIATION OF AMERICAN MEDICAL COLLEGES.

While the 1902 meeting of the Association of American Medical Colleges at Saratoga did not make any radical changes in the requirements for admission to schools, or in an advanced curriculum, there was abundant evidence that at the next meeting advanced requirements in both particulars will be earnestly insisted upon by all the best schools of the Association, and schools that expect to be recognized by State examining boards cannot afford to oppose these measures.

*The Association adhered to its previous requirements of giving no student credit for work done or giving advanced standing except upon rigid examinations.* This should be insisted upon by all State examining boards, for otherwise evidence of higher medical education will be wanting. It is absurd for any one to claim that a student should be given advanced standing, or allowed to pass from one school to another in advanced standing, without having been thoroughly examined by the school in which he received his previous instruction. The practice of giving students credit for attendance upon a course of lectures who have never been examined is obsolete, and in our four years' graded system cannot be tolerated. We must adhere to university principles, and never allow a student to pass from one year to another until he has passed satisfactory examinations, except he may be conditioned in one or two branches, which may be disposed of at the beginning of the next year.

The President of the Association, Dean Vaughn, of the University of Michigan, made timely recommendations for changes in the constitution and by-laws that will elevate the standard of medical education both as to requirements for admission and the curriculum, and also called attention to the pernicious practice of some schools in not conforming to their advertised requirements and in trying to induce students to attend their sessions by offering reduction of fees and other irregular and disreputable inducements.

A committee composed of Dr. Ritchie, dean of the medical department of the University of Minnesota; Dr. Wathen, dean of the Kentucky School of Medicine, and Dr. Dodson, dean of Rush Medical College, was appointed to report at the next meeting of the Association upon the recommendations in the President's Address, and any other reforms they deem in the interest of medical education. A better committee could not have been appointed to do this work, for each of these gentlemen is thoroughly acquainted with the best methods conducive to higher medical education and the honorable relations between medical colleges, and represent schools that will sustain them in any laudable effort in that direction. It can be clearly seen that the Association and State examining boards will but little longer recognize schools that do not literally adhere to the requirements of the Association and the advertised requirements in their catalogues, or such schools as allow students credit for a course of lectures who have attended but part of the same and who have not been examined for advanced standing, or schools that advertise one schedule of fees and then try to induce students to attend by making liberal reductions or issuing scholarships. These schools are disreputable and should not be recognized; but the most disreputable school is the one that offers a reduction of fees to induce students to leave another school, such practice being no better than a moral theft.—*American Practitioner and News.*

